# Integrated Transcriptomic and Histological Analysis of TP53/CTNNB1 Mutations and Microvascular Invasion in Hepatocellular Carcinoma

**DOI:** 10.3390/genes17020190

**Published:** 2026-02-03

**Authors:** Ignacio Garach, Nerea Hernandez, Luis J. Herrera, Francisco M. Ortuño, Ignacio Rojas

**Affiliations:** Department of Computer Engineering, Automation and Robotics, CITIC, University of Granada, 18071 Granada, Spain; igarachv@ugr.es (I.G.); nereahc@ugr.es (N.H.);

**Keywords:** hepatocellular carcinoma (HCC), liver cancer, *TP53*, *CTNNB1*, vascular invasion, RNA-seq, WSI

## Abstract

**Background/Objectives**: Hepatocellular carcinoma (HCC) shows marked molecular and histopathological heterogeneity. Among the alterations most strongly associated with clinical outcome are mutations in *TP53* and *CTNNB1*, as well as the presence of microvascular invasion (MVI). Although these factors are well established as prognostic indicators, how their molecular effects relate to tumor morphology remains unclear. In this work, we studied transcriptomic changes linked to *TP53* and *CTNNB1* mutational status and to MVI, and examined whether these changes are reflected in routine histology. **Methods**: RNA sequencing data from HCC samples annotated for mutations and vascular invasion were analyzed using differential expression analysis combined with machine learning-based feature selection to characterize the underlying transcriptional programs. In parallel, we trained a weakly supervised multitask deep learning model on hematoxylin and eosin-stained whole-slide images using slide-level labels only, without spatial annotations, to assess whether these features could be inferred from global histological patterns. **Results**: Distinct gene expression profiles were observed for *TP53*-mutated, *CTNNB1*-mutated, and MVI-positive tumors, involving pathways related to proliferation, metabolism, and invasion. Image-based models were able to capture morphological patterns associated with these states, achieving above-random discrimination with variable performance across tasks. **Conclusions**: Taken together, these results support the existence of coherent biological programs underlying key risk determinants in HCC and indicate that their phenotypic effects are, at least in part, detectable in routine histopathology. This provides a rationale for integrative morpho-molecular approaches to risk assessment in HCC.

## 1. Introduction

Hepatocellular carcinoma (HCC) is the most common primary malignant tumor of the liver and one of the leading causes of cancer mortality worldwide. Despite advances in clinical treatment, HCC remains a biologically heterogeneous disease, in which tumors of similar stage and histological grade often exhibit very different clinical behavior [1].

One way to differentiate between the different subtypes of HCC is based on different genetic mutations. For example, *TP53* and *CTNNB1* mutations are among the most common molecular alterations in HCC and are associated with specific tumor phenotypes. Inactivating mutations in the *TP53* tumor suppressor gene occur in approximately 30–50% of cases worldwide (with higher rates in regions exposed to aflatoxin B1) [2,3]. These mutations disrupt cell cycle control, DNA repair, and apoptosis, often leading to increased proliferation and a more aggressive phenotype associated with poorer outcomes. The p53 protein, encoded by the *TP53* gene, serves as a guardian of genomic integrity by responding to diverse cellular stresses, including oncogene activation and metabolic disturbances. It manages key protective mechanisms such as cell cycle arrest, DNA repair, and senescence and apoptosis, preventing malignant transformation. Extensive research has established that *TP53* dysregulation, primarily through mutations, drives tumorigenesis in over half of human cancers [4]. On the other hand, mutations in *CTNNB1* (encoding β-catenin) primarily in exon 3, are observed in about 25–40% of HCCs (typically higher in non-viral or alcohol-related cases) [5,6]. These lead to aberrant Wnt/β-catenin pathway activation, promoting metabolic reprogramming, immune exclusion, and a generally less aggressive and better differentiated profile compared to *TP53*-mutated tumors [7]. Notably, *TP53* and *CTNNB1* mutations tend to be mutually exclusive, contributing to separate molecular subclasses and clinical features [8].

In addition to mutational status, vascular invasion is a potential pathological feature associated with poor prognosis in HCC [9,10]. Its presence is strongly correlated with early recurrence after resection or transplantation [11,12]. Although vascular invasion is assessed in surgical specimens, there is growing evidence to suggest that it reflects broader biological programs related to invasion and proliferation, rather than a purely focal process [13].

Genomic and transcriptomic alterations in HCC have been extensively characterized, but their relationship to routine histology remains unclear. Although somatic mutations act through downstream changes in gene expression, it is not always clear how these processes translate into consistent patterns at the tissue level. In practice, it remains unclear whether clinically relevant features, such as microvascular invasion, can be reliably inferred from transcriptomic data or standard hematoxylin and eosin (H&E)-stained preparations.

In this study, we present an integrated morpho-molecular approach to refine HCC risk stratification. Beyond identifying prognostic gene signatures, we frame our analysis around a unifying question: to what extent do distinct molecular alterations and pathological features leave detectable, global morphological signatures in routine histology?

To answer this, we first performed an extensive analysis of differential gene expression on *TP53*/*CTNNB1* activation status and tried to gain an understanding of the profound molecular mechanisms regulating mutated HCCs. In a previous study, we established a 15-gene panel pairing differential gene expression with machine learning that accurately classified liver cancer stages [14]. Building on this work, we applied an identical feature selection procedure to derive a transcriptomic signature for vascular invasion. Subsequently, we trained weakly supervised multitask deep learning models on whole-slide images (WSI) using only slide-level labels. This experimental design allows us to determine if the downstream phenotypic effects of these molecular and focal states are sufficiently pervasive to be inferred from global histological patterns without local annotations.

## 2. Materials and Methods

### 2.1. Data Resource

The Cancer Genome Atlas (TCGA) represents a comprehensive, open-access repository containing extensive multiple omics and clinical information from numerous cancer types [15]. Initiated in 2006 as a joint effort by the National Cancer Institute and the National Human Genome Research Institute in the United States, TCGA has significantly advanced the field of oncology. It has generated critical knowledge about the underlying molecular alterations in cancer, facilitating the identification of therapeutic targets and potential biomarkers for diagnosis and prognosis. Despite the end of the TCGA project, its datasets continue to be available to the scientific community via the Genomic Data Commons (GDC) platform [16], which was the source of data we used in the present study.

**Figure 1:**
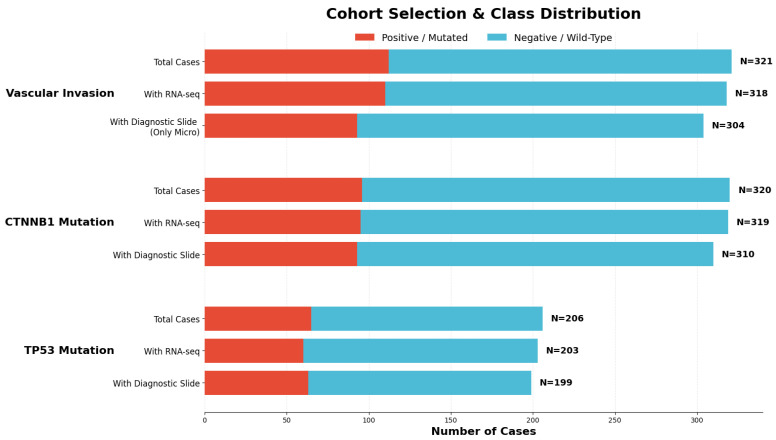
Cohort selection and class distribution from the TCGA-LIHC cohort.

Specifically, WSIs and RNA-Seq gene expression data of hepatocellular carcinoma samples were obtained from the TCGA-LIHC cohort. The cases presenting Single Somatic Mutations (SSMs) on the *TP53* and *CTNNB1* genes were obtained using the *Mutation Frequency* tool. Only coding variants predicted to be functionally damaging were considered. Specifically, variants annotated as possibly damaging or probably damaging by PolyPhen and classified as coding sequence alterations (e.g., missense or stop-gained variants) were retained, while benign or non-coding variants were excluded. Vascular invasion status was retrieved filtering by *Pathology Details Vascular Invasion Type* and selecting *micro* and *macro* statuses. Figure 1 displays the number of samples annotated for each condition. The retrieved files included the samplesheet and clinical data to ensure all samples were correctly identified.

For the histological image classification task, all analyses were performed exclusively on diagnostic (DX) whole-slide images corresponding to formalin-fixed paraffin-embedded (FFPE) tissue sections. Frozen section slides were excluded from this study. This choice was motivated by the higher and more consistent histomorphological quality of FFPE slides, which are known to be better suited for deep learning-based image analysis. In this task, only microvascular invasion cases were considered positive, while macrovascular invasion was excluded due to its low frequency and distinct morphological characteristics, which could confound the detection of subtle invasion-related patterns.

### 2.2. Methodology

#### 2.2.1. Differential Expression and Gene Signature Extraction

Gene-level expression quantification and annotation were performed using the Knowseq package [17]. A quality assessment was carried out with Knowseq (including the Kolmogorov–Smirnov test, Median Absolute Deviation, and distance-based outlier detection), which led to the identification and removal of an outlier. Finally, batch effects were corrected using surrogate variable analysis [18].

Differentially expressed genes (DEGs) were identified using the *limma* package in R, which employs a moderated t-test based on an empirical Bayes approach. This method shrinks the gene-wise residual variances towards a common value, increasing statistical power and stability [19].

For each of the three comparisons considered in the study, separate models were fitted. Log2 fold changes and associated *p*-values were computed, followed by false discovery rate (FDR) adjustment using the Benjamini–Hochberg procedure. Genes were considered differentially expressed using the fold change thresholds and *p*-value cutoffs defined for each contrast in the following Table 1.

In the case of the vascular invasion contrast, a comprehensive machine learning pipeline was implemented using the Knowseq framework to develop a predictive gene signature. All the steps were carried out inside a 5-fold cross-validation procedure, starting with the DEG Extraction. Then, feature selection was performed using the previously extracted DEGs with the minimum redundancy maximum relevance (mRMR) algorithm [20], which generated five rankings. These rankings were used to train SVM models, iteratively adding the ranked genes. Using this, we can have an estimation of the performance of the model for each number of genes. The most frequently selected genes across the five folds were retained as the final proposed signature. To assess its performance, a 100-times repeated final 5-fold cross-validation was conducted. A support vector machine (SVM) was used as a classifier [21] and hyperparameter tuning was performed within each training fold using an inner cross-validation loop to avoid overfitting [22].

#### 2.2.2. WSI Classification

**Figure 2:**
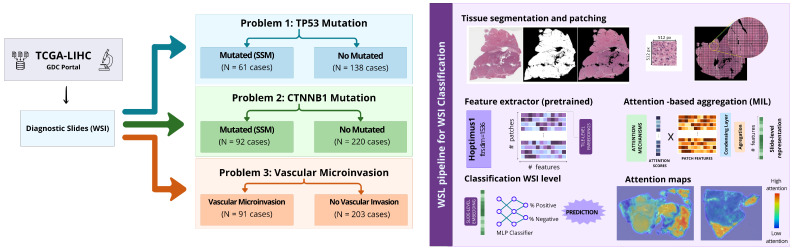
Weakly supervised learning (WSL) pipeline for whole-slide image (WSI) classification. The pipeline comprises four key stages: (1) tissue segmentation and preprocessing, (2) patch tessellation and feature extraction, (3) MIL-based feature aggregation, and (4) slide-level classification with interpretability support (Red indicates high attention, and blue indicates low attention).

The methodological workflow of this study integrates whole-slide histopathology images with weakly supervised deep learning to explore the relationship between tissue morphology and molecular alterations in hepatocellular carcinoma. An overview of the main processing steps, including image preprocessing, feature extraction, multiple instance learning, and evaluation, is provided below and illustrated in Figure 2.

1. Preprocessing and Tissue Segmentation

To minimize computational demands and prioritize diagnostically relevant areas, we applied background filtering. Tissue regions were identified by generating a binary mask via Otsu’s thresholding [23], which optimally separates foreground (tissue) from background (empty glass or whitespace) using pixel intensity histograms.

Valid tissue areas were then divided into non-overlapping 512 × 512 pixel patches at 40× magnification, corresponding to an effective spatial resolution of approximately 0.25 microns-per-pixel, as obtained from WSI metadata. Patches with insufficient tissue coverage were filtered out to maintain high-quality inputs.

2. Feature Extraction

Patch-level features were extracted using a pre-trained foundation model tailored for histopathology. Specifically, we employed weights from the Bioptimus H-optimus1 [24] model (a Vision Transformer-based architecture with over 1 billion parameters, pre-trained via self-supervised learning on a large-scale proprietary dataset of hundreds of millions of histopathology images from diverse patients and diseases).

Formally, for a WSI *W*, we derive a bag of *N* patches $X=\{x_{1},x_{2},\ldots ,x_{N}\}$. The feature extractor f(·) maps each patch $x_{i}$ to a fixed-dimensional embedding $h_{i}\in R^{D}$:

$$h_{i}=f\left(x_{i}\right)$$

This yields a compact bag of embeddings $H=\{h_{1},\ldots ,h_{N}\}$, reducing dimensionality while retaining essential morphological details.

3. Attention-Based MIL Aggregation

Given weak supervision (slide-level labels only), we evaluated several attention-based MIL methods to aggregate instance embeddings *H* into a single slide-level representation *Z* in a permutation-invariant manner. The compared aggregators include the following:

- ABMIL (Attention-Based MIL) [25]: Employs a gated attention mechanism to assign learnable importance weights to individual instances, enabling the model to focus on the most informative patches while preserving permutation invariance.
- TransMIL (Transformer-Based MIL) [26]: Utilizes a Transformer architecture to model global contextual relationships among instances, capturing long-range dependencies between patches within a slide.
- DFTD (Dual-Feature Tokenization MIL) [27]: Decomposes each bag into complementary global and local feature representations, enabling the model to identify informative instances through interactions between discriminative features and contextual information, without explicitly constraining attention via clustering.
- CLAM (Clustering-Constrained Attention MIL) [28]: Introduces instance-level clustering to explicitly separate positive and negative evidence within a bag, guiding the attention mechanism toward discriminative regions.

4. Slide-Level Classification

The aggregated representation *Z* is passed to a multi-layer perceptron (MLP), which projects it into the label space. Predictions are produced via softmax (multi-class) or sigmoid (binary) activation.

5. Interpretability and Visualization

For model explainability, attention weights from the MIL aggregator were visualized as heatmaps. Normalized scores $a_{i}$ were overlaid on the original WSI coordinates, highlighting regions of interest (ROIs) most influential to the prediction. This facilitates pathologist review and qualitative assessment of model decisions.

6. Evaluation Criteria

Due to the limited number of samples in the minority class (≈60 WSI), we adopted a 5-fold cross-validation protocol over the full dataset to maximize data utilization and obtain a stable performance estimate. All models were evaluated using the same cross-validation folds, with fixed hyperparameters, and each WSI was used exactly once for testing. To address class imbalance, training employed a class-weighted cross-entropy loss, and model selection within each fold was based on the F1-score of the positive class.

## 3. Results and Discussion

### 3.1. *TP53* Mutations

#### 3.1.1. Differential Expression Analysis

Following the described preprocessing steps, a total of 249 statistically significant differentially expressed genes (DEGs) were identified, of which 123 were upregulated and 126 downregulated in *TP53*-mutated hepatocellular carcinoma compared to the wild-type group (Table 2).

One of the most direct transcriptomic consequences of *TP53* mutation is the silencing of genes that strictly depend on p53 for their expression. Our analysis reveals a cluster of significantly downregulated genes, *PPP1R7*, *EDA2R*, *PURPL*, *PTCHD4*, *HGFAC*, and *SPATA18*, representing the collapse of critical surveillance mechanisms governing mitosis, apoptosis, and metabolism.

Among these, *EDA2R* is a direct transcriptional target of p53 [29]. Its signaling is predominantly involved in anoikis (cell death induced by detachment from the extracellular matrix). In normal epithelial cells, loss of anchorage triggers p53-dependent *EDA2R* expression, ensuring that displaced cells die rather than colonize inappropriate sites [30,31]. However, in *TP53*-mutated HCC cells, the failure to induce *EDA2R* may contribute to tumor cell survival in suspension or after detachment from the basement membrane. This anoikis resistance seems to have an important role in intrahepatic metastasis and systemic dissemination.

A similar tumor-suppressive role is observed for *SPATA18*, which encodes a p53-inducible protein that induces lysosome organelles within mitochondria, eliminating oxidized mitochondrial proteins and functioning as a tumor suppressor via mitochondrial quality control [32]. In both renal cell carcinoma and colorectal cancer, elevated *SPATA18* expression has been associated with improved prognosis and overall survival [32,33], which further supports the observation that *TP53*-mutant tumors exhibit poorer prognosis in association with reduced *SPATA18* expression.

Metabolic dysregulation is further highlighted by the downregulation of *HGFAC*, a serine protease that activates Hepatocyte Growth Factor (HGF). It is a marker of mature, functional hepatocytes. Beyond HGF activation, it maintains systemic glucose and lipid homeostasis [34]. The downregulation of this gene may exacerbate lipid dysregulation, enabling rapid cell proliferation, something that has previously been linked to the lipogenic phenotype of aggressive cancers [35]. Moreover, liver cancer patients with low *HGFAC* expression, linked to DNA hypermethylation, have been found to present significantly poorer survival than those with high expression [36,37].

In the same metabolic context, *CYP2E1* is a key enzyme involved in ethanol and fatty acid metabolism. It has been found to be consistently downregulated in poorly differentiated HCC, and its expression correlated negatively with tumor size and vascular invasion [38]. Its loss in established tumors may protect cancer cells from excessive oxidative stress-induced death, contributing to the more aggressive phenotype of *TP53*-mutated HCC.

Finally, the significant downregulation of asialoglycoprotein receptors (*ASGR1* and *ASGR2*) in *TP53*-mutated HCC has been suggested to signify a critical event of *functional dedifferentiation*, where tumor cells progressively lose their specific hepatocellular identity to acquire a more invasive phenotype [39]. Under healthy conditions, ASGRs are abundantly expressed on the surface of mature hepatocytes, functioning as a clearance system for desialylated glycoproteins [40]. However, in aggressive *TP53*-mutated tumors, the suppression of this system is consistent with a metastatic phenotype, potentially facilitating cell migration [41]. Again, this downregulation relates *TP53*-mutated cases with the most aggressive phenotypes of the disease.

In contrast to these losses of tumor-suppressive and differentiation programs, the specific upregulation of GABAergic signaling components (*GABRA2* and *GABRA3*) observed in *TP53*-mutated HCC aligns with the emerging concept of *neuronal mimicry*, where aggressive tumors hijack neuronal pathways to support survival and progression [42,43]. While GABA typically works as the primary inhibitory neurotransmitter in the mature adult brain, it reverts to an excitatory and proliferative role in neoplastic tissues, mirroring the physiology of embryonic neural stem cells [44,45].

#### 3.1.2. Gene Ontology Enrichment

**Figure 3:**
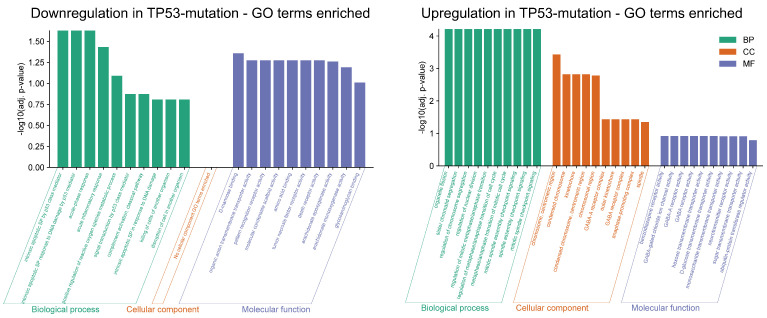
*TP53* DEGs Gene Ontology enrichment analysis.

The top Gene Ontology (GO) terms associated with the DEGs provide a summary of the biological shifts occurring in *TP53*-mutated HCC (see Figure 3).

At the level of biological processes, the most clear signature is a profound dysregulation of the cell cycle. This enrichment is driven by the strong upregulation of key mitotic regulators, including *TOP2A*, *BUB1B*, *CDCA8*, *CDC20*, and *CCNB1* [46]. These genes collectively orchestrate the G2/M transition and faithful chromosome segregation. Their overexpression in the context of *TP53* mutation reflects the collapse of the p53-p21-DREAM pathway [47]. The resulting *mitotic storm* is indicative of accelerated proliferation and may foster chromosomal instability.

Similarly, enrichment in the regulation of the apoptotic process underscores the evasion of apoptosis. This term is influenced by the downregulation of proapoptotic genes such as *EDA2R*, *SPATA18*, and *PPP1R7*, jointly with the functional loss of *TP53* in the mutated group. The combined effect seems to disable apoptotic safeguards, allowing cells with severe mitotic defects to survive and propagate.

Kinetochore and spindle-associated terms are enriched in the cellular component analysis, driven by *BUB1B*, *CDCA8*, and *PPP1R7*, that collectively disrupt accurate chromosome alignment and segregation [48,49,50]. This dysregulation directly contributes to aneuploidy, which is a common feature of *TP53*-mutated tumors and a driver of intratumoral heterogeneity and poor prognosis [51].

Finally, the molecular function category reveals targeted enzymatic and ion channel reprogramming. GABA-A receptor activity reflects the functional gain of chloride conductance via *GABRA2* and *GABRA3*, potentially altering membrane potential and intracellular signaling in a manner advantageous to tumor cell survival.

#### 3.1.3. Slides

Next, we evaluated the ability of weakly supervised deep learning to predict *TP53* mutation status directly from whole-slide images of hepatocellular carcinoma (LIHC). Several attention-based multiple instance learning (MIL) models were assessed using slide-level labels only, without any spatially localised annotations. All models were trained and evaluated under the same 5-fold cross-validation protocol on the TCGA-LIHC cohort. As reported in Table 3, CLAM achieved the highest ROC-AUC, while ABMIL showed slightly higher sensitivity and comparable F1-score for the *TP53*-mutated class. Based on its overall performance, CLAM was selected for further qualitative analysis.

For each fold, the model was trained on four subsets and evaluated on the remaining one. Across the five folds, CLAM achieved a mean ROC-AUC of 0.82 ± 0.07 and a mean accuracy of 0.77 ± 0.07. Although some variability was observed across folds, performance remained relatively consistent in the identification of *TP53*-mutated cases.

**Figure 4:**
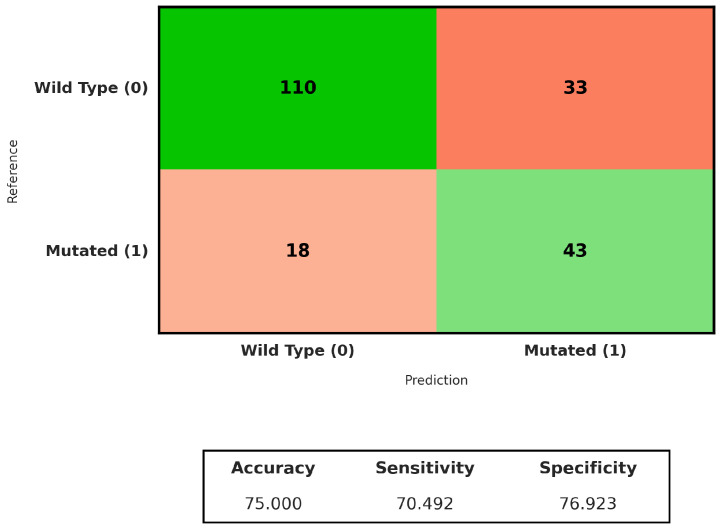
Confusion matrix of the CLAM model for *TP53* mutation prediction at the whole-slide level.

The aggregated confusion matrix shown in Figure 4 indicates that 112 out of 143 *TP53* wild-type cases were correctly classified, corresponding to a specificity of 77%. For the *TP53*-mutated class, 43 out of 61 cases were correctly identified, yielding a sensitivity of 70%. These results indicate that a substantial fraction of *TP53* mutations can be inferred from histomorphological patterns alone under a weakly supervised setting; however, a subset of mutated tumors remains difficult to detect in the absence of region-level annotations.

Importantly, these predictions were obtained using weak supervision only, without any spatial annotation of tumor regions. This suggests that attention-based MIL models are able to capture global morphological correlates of molecular alterations from whole-slide images. The reduced sensitivity observed for *TP53*-mutated cases is consistent with the heterogeneous phenotypic manifestations associated with *TP53* alterations in hepatocellular carcinoma, indicating that not all mutations give rise to clearly distinguishable histomorphological patterns.

**Figure 5:**
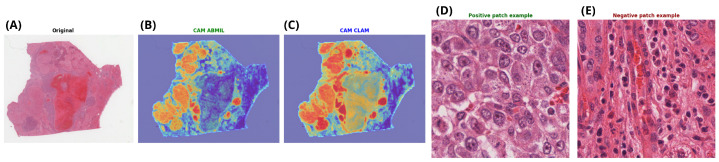
Representative example of *TP53* mutation prediction at the whole-slide level. (**A**) Original H&E-stained whole-slide image. (**B**,**C**) Attention heatmaps generated by the two best-performing models (CLAM and ABMIL), highlighting regions contributing most to the slide-level prediction (Red indicates high attention, and blue indicates low attention). (**D**) Patch with the highest positive attention score, corresponding to strong evidence for *TP53* mutation. (**E**) Patch with the lowest attention score, representing negative evidence.

As illustrated in Figure 5, attention maps derived from both ABMIL and CLAM highlight spatially coherent tumor regions rather than isolated patches. Representative high-attention patches tend to exhibit increased cellular density and nuclear atypia, whereas low-attention regions often correspond to more structured or stromal areas. These observations support the ability of attention-based MIL models to capture biologically meaningful morphological patterns under weak supervision.

### 3.2. *CTNNB1* Mutation

#### 3.2.1. Differential Expression Analysis

Using the *CTNNB1* annotation, we repeated the DEGs extraction process. In this contrast, 245 DEGs were identified, of which 152 were upregulated and 93 downregulated in the *CTNNB1*-mutated group. The results are consistent with the Wnt/β-catenin pathway activation, revealing coordinated effects on signaling and metabolism and suggesting some molecular mechanisms of the typical immune exclusion and differentiation characteristics of the *CTNNB1*-mutant phenotype (Table 4).

Among the top upregulated genes, we found *NKD1*, a negative feedback regulator and transcriptional target of Wnt signaling activation in liver and intestinal tumors, and suspected to enhance cancer proliferation [52,53].

Another upregulated gene, *REG3A*, is a C-type lectin expressed in the intestine and pancreas but inducible in the liver during injury [54]. It has been identified as a direct target of β-catenin, linking Wnt activation to regenerative and survival programs. Its upregulation suggests that *CTNNB1*-mutated tumors exploit normal liver regeneration pathways to enable growth under oxidative or toxic stress. The clinical implications of *REG3A* expression appear to be context dependent. In hepatocellular carcinoma, *REG3A* has been associated with early-stage tumors without vascular invasion and a more favorable prognosis [55]. In contrast, high *REG3A* expression in colorectal cancer has been linked to enhanced proliferative ability and poorer patient outcomes [56], suggesting that in non-regenerative contexts *REG3A* may contribute directly to oncogenic progression [54].

We also observed strong induction of *ODAM*, a recently described and highly specific Wnt/β-catenin target in liver cancer [57]. *ODAM* is typically not expressed in normal liver tissue but is greatly induced in *CTNNB1*-mutated HCC, making it almost diagnostic of aberrant Wnt activation [58]. Its detection in this cohort provides confirmation of this novel angle for stratifying patients with Wnt-driven disease. At the same time, *ODAM* expression has shown to improve apoptosis in breast cancer cells, suppress growth rate, and migratory activity [59]. These previous reports suggest a similar mechanism in *CTNNB1*-mutated HCC, given its typical less aggressive phenotype.

A defining feature of *CTNNB1*-mutated HCC is its distinct metabolic phenotype, resembling that of pericentral hepatocytes (where HCC typically arises), driving the expression of genes involved in glycolysis, xenobiotic metabolism, and lipogenesis [60,61]. Consistent with this model, *CYP2E1*, the canonical marker of Zone 3 hepatocytes, was strongly upregulated [60]. *CYP2E1* activity generates high levels of reactive oxygen species (ROS), placing these tumors under oxidative stress. Tumor survival under these conditions often requires co-activation of antioxidant pathways [62]. This metabolic configuration correlates with the well-differentiated histological appearance typical of this subclass [63]. In line with elevated oxidative stress, *ALDH3A1* was also upregulated. It has been explicitly identified as another marker of Wnt/β-catenin activation in hepatocellular adenomas and carcinomas, and its overexpression likely represents an adaptation that may buffer against apoptosis in a high-ROS environment. Elevated ALDH activity has also been associated with intrinsic resistance to alkylating chemotherapeutic agents [64,65], suggesting a target for potential treatments specific for this subclass.

*CTNNB1*-mutated HCCs are typically classified as immunologically cold tumors, showing a suppressed immune microenvironment with reduced infiltration of effector cells and poor response to checkpoint inhibitors [66]. Some of the found DEGs are related to these mechanisms.

The found downregulation of *CASP1* likely plays a central role in suppressing immune danger signals by inhibiting the inflammasome pathway. Under normal conditions, this pathway activates pro-inflammatory cytokines that initiate pyroptosis—a form of highly immunogenic cell death that promotes T-cell recruitment [67,68]. In *CTNNB1*-mutated HCCs, Wnt/β-catenin signaling appears to mediate this suppression, elevating the threshold for pyroptosis and shifting toward non-immunogenic apoptosis or enhanced cell survival under stress, evading immune surveillance [69]. Additionally, we observed downregulation of *CXCL1* and *CXCL6* chemokines, which in wild-type tumors attract neutrophils and initiate innate immune response [70]. Clinically, this aligns with the resistance to therapies like transarterial chemoembolization, suggesting they are not a good option for patients with *CTNNB1*-mutation.

#### 3.2.2. Gene Ontology Enrichment

**Figure 6:**
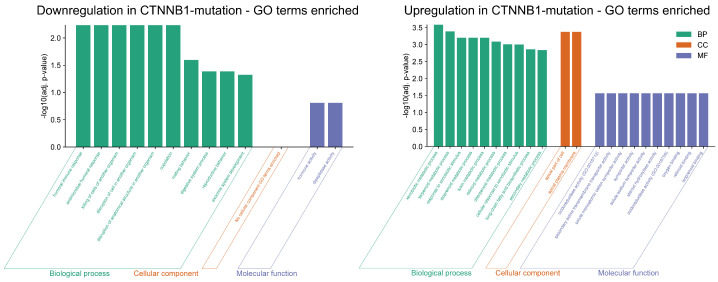
*CTNNB1* DEGs Gene Ontology enrichment analysis.

At the level of biological processes (Figure 6), we observed a profound metabolic reprogramming. This enrichment is driven by the upregulation of key metabolic enzymes and regulators, including *CYP1A1, CYP1A2, CYP2E1, CYP3A4, UGT1A7, UGT1A9, SLC22A11, SLC22A12*, and *ACSL6*. These genes collectively facilitate shifts in amino acid biosynthesis, fatty acid metabolism, glutamate processing, and glycolysis. Their overexpression in the context of *CTNNB1* mutation reflects activation of the Wnt/β-catenin pathway [47], promoting adaptive metabolic flexibility that supports tumor cell proliferation and survival under stress. The resulting metabolic storm enables efficient energy production and resource allocation even under the harsh tumor microenvironment.

The cellular component ontology is exclusively defined by terms related to the apical domain of the cell, specifically the apical plasma membrane. In polarized hepatocytes, the apical membrane forms the bile canaliculi [71], which are essential for secreting metabolized and detoxified compounds (xenobiotics, and bile acids) into the biliary system [72]. The strong enrichment for this component supports and physically localizes the identified biological processes; the metabolic activities are functionally linked to the apical secretory apparatus. In the context of the *CTNNB1* mutation, this may indicate an attempt by the tumor cells to maintain this critical detoxification function.

The molecular functions reveal a toolkit for metabolic transformation and transport. This includes oxidoreductase, an enzyme essential for detoxifying xenobiotics and metabolizing lipids, alongside various transporters that improve the movement of metabolites across cells. These functions integrate with the metabolic reprogramming observed in the biological processes, enabling the efficient handling and secretion of compounds through the apical plasma membrane. This reinforces the *CTNNB1* tumor’s detoxification capabilities, potentially contributing to drug resistance.

#### 3.2.3. Slides

After analyzing *TP53* mutation status, we used the same weakly supervised framework to predict *CTNNB1* mutations in hepatocellular carcinoma. We evaluated multiple attention-based MIL models under identical experimental conditions. As shown in Table 5, ABMIL achieved the highest overall performance, with results comparable to those obtained with CLAM.

The best model had an overall accuracy of 0.73 and an ROC-AUC of 0.79. Predictive performance was slightly lower than that observed for *TP53*. The difference was modest. This indicates that *CTNNB1*-associated alterations may also be reflected in histomorphological patterns detectable in routine H&E whole-slide images. However, these alterations are less consistent.

The discrepancy in performance between *TP53* and *CTNNB1* is biologically plausible and likely reflects the distinct functional roles of the two genes. At the histological level, pronounced nuclear atypia and architectural disruption are readily apparent features of *TP53* inactivation. On the other hand, *CTNNB1* mutations mostly trigger the Wnt/β-catenin signaling pathway and are often linked to more subtle and varied morphological characteristics, such as trabecular growth patterns, focal cholestasis, or relatively preserved differentiation. These characteristics are not evenly spread across all mutated tumors.

**Figure 7:**
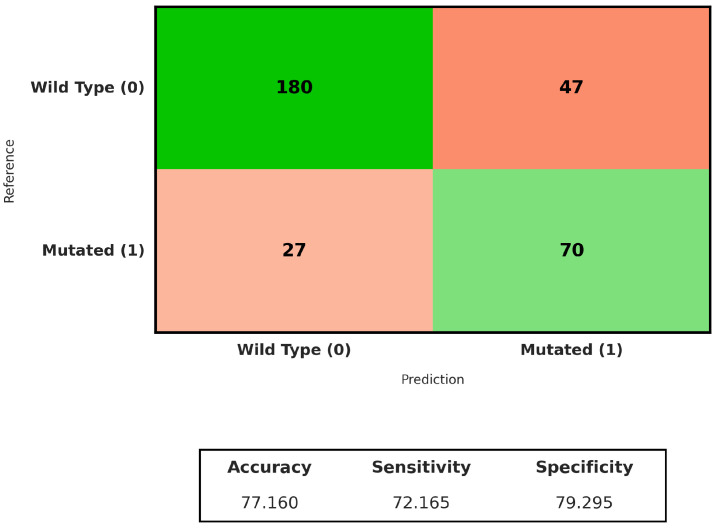
Confusion matrix of the ABMIL model for *CTNNB1* mutation prediction at the whole-slide level.

The aggregated confusion matrix (Figure 7) shows that 180 out of 227 *CTNNB1* wild-type slides were correctly classified, corresponding to a specificity of 79%. For the *CTNNB1*-mutated class, 70 out of 97 cases were correctly identified, yielding a sensitivity of 72%. Most errors arose from mutated cases misclassified as wild-type, consistent with the more heterogeneous and less distinctive histomorphological presentation associated with *CTNNB1* alterations.

**Figure 8:**
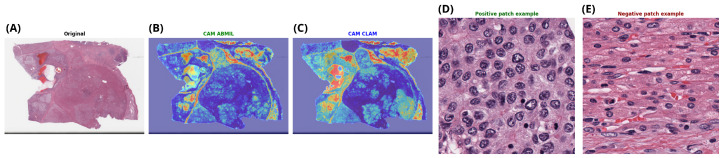
Representative example of *CTNNB1* mutation prediction at the whole-slide level. (**A**) Original H&E-stained whole-slide image. (**B**,**C**) Attention heatmaps generated by the two best-performing models (CLAM and ABMIL), highlighting regions contributing most to the slide-level prediction (Red indicates high attention, and blue indicates low attention). (**D**) Patch with the highest positive attention score, corresponding to strong evidence for the *CTNNB1* mutation. (**E**) Patch with the lowest attention score, representing negative evidence.

In line with the findings for *TP53*, attention maps for *CTNNB1* (Figure 8) prediction showed a high degree of agreement between the best-performing models. Regions assigned high attention scores were spatially coherent across methods, while negatively weighted patches corresponded to areas with clearly different histological appearances. This qualitative consistency supports the notion that, despite the lower overall performance, *CTNNB1*-associated morphological cues are not random but are captured in a reproducible manner by attention-based MIL models.

### 3.3. Vascular Invasion

#### 3.3.1. Gene Signature Identification via Machine Learning

We will now categorize the presence of vascular invasion in the tumors, again by gene expression values. In this case, we have integrated the DEG Extraction process into a 5-fold cross validated machine learning pipeline. For each training fold of the cross-validation procedure, we extracted DEGs (139/142/135/134/139 were found, 102 common across folds) and applied the mRMR algorithm to them, obtaining an importance ranking. Then, adding genes iteratively in the ranking obtained by the algorithm, SVM classifiers were trained and tested in the samples of the remaining fold.

**Figure 9:**
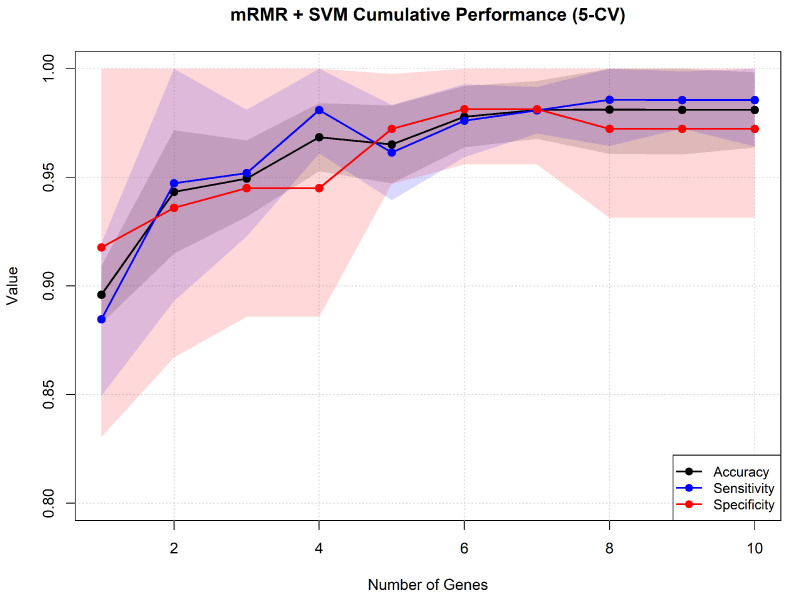
Evolution of cross-validation classification metrics across the 5-fold mRMR rankings. Solid lines denote mean performance, while the shaded colored areas indicate the standard deviation across the folds.

Figure 9 displays the mean accuracies, sensitivities, and specificities, paired with the standard deviation across folds for each incremental gene set. As the evolution reaches stability with four genes (mean 96.85% accuracy), we selected it as a compromise between complexity and performance. Using the rankings obtained by mRMR across folds, we selected the most robust genes.

The proposed final signature comprises *PGM5P4-AS1* (ranked 1st in 3 folds), *IRX3* (appeared in the top 3 in 4 folds), *LINC00683* (ranked 1st in 2 folds), and *AC112206.3* (consistently in the top 10).

To estimate the performance of the selected signature, a new 100-times repeated, 5-fold cross-validation was performed. This model achieved an accuracy of 97.81%±1.68, a sensitivity of 96.98%±3.66, and a specificity of 98.25%±1.88.

While the cross-validation strategy provides an estimation of performance, we must interpret these high metrics with caution. Given the modest sample size, imbalance and the high-dimensionality, we acknowledge the potential risk of overfitting. Although the internal cross-validation mitigates this to an extent, it cannot rule out cohort-specific effects.

Moreover, the final fixed signature was determined by analyzing consensus rankings across the entire dataset, which introduces selection bias and prevents it from serving as an unbiased estimate of predictive performance. However, this approach is valuable because it prioritizes genes that consistently rank high across different data partitions, thereby filtering out stochastic noise and artifacts specific to individual subsets. It also yields a concrete panel that can be targeted and verified in future external validation studies.

Consequently, the reported performance represents a promising initial indication of the signature’s potential, while true generalizability requires further validation on larger external cohorts to address selection bias and confirm its utility.

The selected gene signature was further supported by the biological relevance of the genes in the literature:

*PGM5P4-AS1* is a long non-coding RNA (lncRNA) that appears to act as a tumor suppressor. In lung cancer, *PGM5P4-AS1* is downregulated in tumors, and its overexpression inhibits cell proliferation and invasion [73]. Although direct studies in HCC or vascular invasion are lacking, *PGM5P4-AS1* downregulation was identified as part of a gallbladder cancer diagnostic signature [74] suggesting it may similarly mark tumor suppression. In our analysis, *PGM5P4-AS1* was found to be downregulated in HCC samples presenting vascular invasion, consistent with the previously reported lung and bladder behavior.

Likewise, *LINC00683* is another lncRNA that seems to act as a tumor suppressor. It is downregulated in prostate cancer and high levels correlate with better survival [75]. In that context, gene-set analysis showed *LINC00683* is involved in cancer pathways such as Wnt/β-catenin signaling. Moreover, *LINC00683* appeared in a multi-RNA signature for cervical cancer recurrence [76]. Together, these suggest *LINC00683* normally restrains oncogenic signaling. These conclusions were also proven in our study, showing that *LINC00683* downregulation could be involved in the promotion of vascular invasion of adjacent tissues.

Although *IRX3* has been shown experimentally to promote proliferation in HCC cell line [77], its underexpression in vascular invasive tumors in our analyzed cohort suggests a context-dependent role. Homeobox transcription factors like *IRX3* are linked to differentiation and early proliferative programs, and their expression can vary significantly with tumor progression stage [78]. It is plausible that vascular invasive HCC represents a later phenotypic state in which alternative signaling pathways lead to relative *IRX3* repression.

Finally, for *AC112206*, the available evidence related to cancer is still scarce. In non-small cell lung cancer, low expression of *AC112206* has been reported to correlate with poorer overall survival [79], suggesting a potential tumor-suppressive role in that context. However, to date there are no clear or direct links between *AC112206* and liver cancer or vascular invasion specifically.

#### 3.3.2. Slides

Once more, in addition to looking at changes in genes, we studied whether models that are not strongly supervised could see signs of vascular microinvasion. Vascular microinvasion is a factor that doctors use to predict how well a patient will do with hepatocellular carcinoma. We evaluated all models using the same experimental setup applied to the mutation prediction tasks. As shown in Table 6, the overall performance was substantially lower than that observed for *TP53* and *CTNNB1*.

Across models, ROC-AUC values ranged between 0.57 and 0.66, with the highest mean AUC (0.66 ± 0.10) being achieved by CLAM. However, no approach showed consistently strong performance across sensitivity and F1-score, indicating limited discriminative power at the whole-slide level.

**Figure 10:**
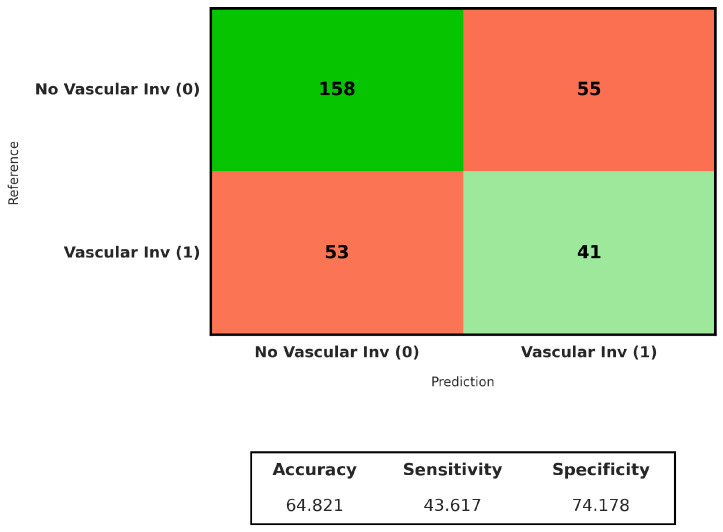
Confusion matrix of the CLAM model for vascular invasion prediction at the whole-slide level.

The aggregated confusion matrix for vascular invasion prediction in Figure 10 shows that 158 out of 213 non-invasive cases were correctly classified, corresponding to a specificity of 74%. For cases with vascular invasion, 41 out of 94 slides were correctly identified, yielding a sensitivity of 44%. Most errors were associated with invasive tumors misclassified as non-invasive, indicating limited discriminative power at the whole-slide level.

This reduced performance is not unexpected. Molecular alterations may influence tumor morphology over large tissue areas, resulting in histological patterns that are broadly distributed across the slide. In contrast, vascular microinvasion is a focal pathological phenomenon, typically confined to small regions at the tumor–vascular interface, which may represent only a minor fraction of the whole-slide image or may not be captured among the sampled tiles at all. This creates a fundamental challenge for the MIL framework used under weak supervision. Because the model relies on attention mechanisms to aggregate features from thousands of tiles into a single slide-level prediction, the informative signal originating from a small number of invasion-positive tiles is easily diluted by the overwhelming number of non-informative background tiles. In the absence of pixel- or region-level annotations to explicitly guide the attention mechanism toward these rare events, the model faces intrinsic difficulty in isolating the relevant morphological events.

The low sensitivity seen in most models supports this idea and suggests that false-negative predictions are probably due to sampling limitations, not the lack of histological correlates. Although some models achieved moderate area under the curve (AUC) values, these results were not generalizable across the cohort, underscoring the heterogeneous and spatially restricted nature of vascular microinvasion in liver hepatocellular carcinoma.

**Figure 11:**
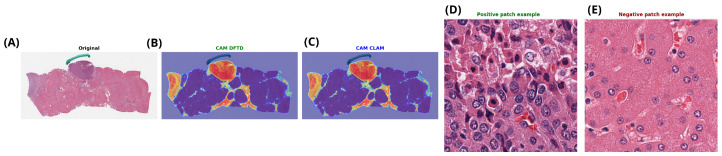
Representative example of vascular invasion prediction at the whole-slide level. (**A**) Original H&E-stained whole-slide image. (**B**,**C**) Attention heatmaps generated by CLAM and ABMIL, highlighting regions contributing most to the slide-level prediction (Red indicates high attention, and blue indicates low attention). (**D**) Patch with the highest positive attention score, corresponding to strong evidence for vascular invasion. (**E**) Patch with the lowest attention score, representing negative evidence.

For vascular invasion, although overall predictive performance was modest across models, the corresponding attention maps in Figure 11 showed consistent spatial patterns. In particular, regions highlighted by different MIL architectures largely overlapped, suggesting that the models converge on similar histomorphological cues despite limited discriminative power at the slide level.

### 3.4. Limitations and Generalizability

Finally, we acknowledge the limitations regarding generalizability inherent to the use of the TCGA-LIHC dataset. While this cohort provides a unique paired morpho-molecular resource, it introduces specific biases in case selection, as it predominantly comprises surgically resectable, treatment-naïve tumors. As a result, the models are trained on a population that may not fully represent the advanced or unresectable HCC cases commonly encountered in routine clinical practice.

Regarding slide preparation, TCGA whole-slide images exhibit substantial heterogeneity in staining protocols and scanning devices across contributing institutions. Although this variability poses challenges for model development, it may also act as a partial proxy for real-world heterogeneity, encouraging the learning of robust, scanner-invariant representations rather than overfitting to institution-specific artifacts. Nevertheless, successful deployment in external clinical settings will require rigorous validation on independent cohorts and, potentially, the application of domain adaptation strategies to address domain shift arising from differences in digitization pipelines and from the transition between resection specimens and diagnostic biopsies.

## 4. Conclusions

We showed that the transcriptomic landscape of *TP53*-mutated HCC is defined by a shift towards a proliferative and dedifferentiated state. These insights reinforce the central role of *TP53* loss in the development of aggressive HCC phenotypes, but also highlight potential therapeutic entry points. Conversely, the *CTNNB1*-mutated group shows a profile dominated by the activation of the Wnt/β-catenin pathway and metabolic reprogramming (displayed by the Gene Ontology Enrichment). This results in a *cold* tumor microenvironment, with downregulated chemokines like *CXCL1* and *CXCL6*, limiting the activation of the immune system. While these tumors seem to suffer from oxidative and toxic stress, they utilize alternative survival strategies—such as pyroptosis inhibition to sustain growth. Additionally, the vascular invasion signature achieved high cross-validation accuracy. While these results are promising, the modest sample size and lack of external validation warrant caution regarding potential overfitting and cohort overspecification. Nonetheless, the consistent selection and performance of genes across folds suggest that they provide relevant information.

At the histological level, WSI-based classification revealed that *TP53* and *CTNNB1* mutation status can be predicted with comparable performance when restricting the analysis to alterations with clear functional impact, suggesting that both mutations leave detectable global morphological traces. In contrast, vascular invasion proved consistently harder to predict, in line with its focal and spatially restricted nature. These results indicate that weakly supervised MIL models can recover biologically meaningful signals from routine histology, but that their performance is inherently constrained by how directly a given molecular or pathological event manifests at the tissue scale.

Revisiting our central research question, our findings clarify the extent to which molecular and pathological features are reflected in routine histology. Molecular alterations associated with widespread cellular reprogramming—such as *TP53* and *CTNNB1* mutations—imprint detectable global signatures that are amenable to slide-level prediction. By contrast, focal pathological phenomena like microvascular invasion induce a resolution mismatch under weak supervision, limiting achievable performance. Consequently, while deep learning offers powerful tools for decoding diffuse molecular states from H&E slides, more granular supervision will likely be required to robustly capture spatially localized events. This distinction is critical for the future development of integrative morpho-molecular biomarkers in HCC.

## Figures and Tables

**Table 1:** Differentially expressed genes identification thresholds and number of DEGs retained for the three comparisons.

Comparison	$\left|\log_{2}\;\mathrm{Fold}\;\mathrm{Change}\right|$	FDR *p*-Value	DEGs Retained
*TP53* Mutant vs. Wild Type	0.8	0.001	249
*CTNNB1* Mutant vs. Wild Type	1.0	0.001	245
Vascular Invasion Presence	0.5	0.001	102 common

**Table 2:** Key differentially expressed genes in *TP53*-mutated HCC.

Gene	LogFC	Mechanistic Association in *TP53* Mutant Context
*PPP1R7*	−2.69	Loss of p53-dependent mitotic control; resistance to apoptosis.
*EDA2R*	−2.61	Failure of p53-mediated anoikis signaling; facilitates metastasis.
*SPATA18*	−1.86	Metabolic reprogramming; poor prognosis.
*HGFAC*	−1.89	Disrupts lipid and glucose homeostasis.
*CYP2E1*	−1.44	Loss of ethanol and fatty acid metabolism; reduces oxidative stress.
*ASGR2*	−1.09	Loss of glycoprotein clearance promotes metastasis.
*GABRA2*	+1.26	GABAergic signaling; proliferation and neuronal mimicry.
*TOP2A*	+0.85	Derepression of the p53–p21–DREAM pathway.
*BUB1B*	+0.84	Aneuploidy and mitotic progression despite errors.

**Table 3:** Comparison of attention-based MIL models for *TP53* mutation prediction. Sensitivity and F1-score correspond to the positive (mutated) class. The best results are marked in bold.

Model	ROC-AUC (±std)	Sensitivity	F1-Score
ABMIL	0.79 ± 0.07	**0.72**	**0.63**
TransMIL	0.73 ± 0.09	0.43	0.46
DFTD	0.75 ± 0.07	0.59	0.52
CLAM	**0.82** ± **0.07**	0.70	**0.63**

**Table 4:** Key differentially expressed genes in *CTNNB1*-mutated HCC.

Gene	LogFC	Mechanistic Association in *CTNNB1* Mutant Context
*CXCL1*	−1.42	Limits neutrophil recruitment; weakens innate immunity.
*CXCL6*	−1.29	Suppresses chemokine-mediated immune cell infiltration.
*CASP1*	−1.02	Inhibition of inflammasome activation; immune escape.
*NKD1*	+2.58	Negative feedback regulator of Wnt/β- signaling; proliferation.
*REG3A*	+2.70	Resistance to apoptosis under oxidative or toxic stress.
*ODAM*	+2.31	Wnt/β-catenin target in liver cancer; less aggressive phenotype.
*CYP2E1*	+2.32	Metabolic reprogramming; increased oxidative stress.
*ALDH3A1*	+2.69	Survival under stress; resistance to alkylating chemotherapies.

**Table 5:** Comparison of attention-based MIL models for *CTNNB1* mutation prediction. Sensitivity and F1-score correspond to the positive (mutated) class. The best results are marked in bold.

Model	ROC-AUC (±std)	Sensitivity	F1-Score
ABMIL	**0.79** ± **0.02**	**0.72**	**0.65**
TransMIL	0.66 ± 0.05	0.47	0.43
DFTD	0.67 ± 0.04	0.56	0.49
CLAM	0.79 ± 0.05	0.65	0.62

**Table 6:** Comparison of attention-based MIL models for vascular invasion prediction. Sensitivity and F1-score correspond to the positive (presence of invasion) class. The best results are marked in bold.

Model	ROC-AUC (±std)	Sensitivity	F1-Score
ABMIL	0.61 ± 0.10	0.52	0.45
TransMIL	0.60 ± 0.08	0.54	**0.47**
DFTD	0.57 ± 0.13	**0.62**	0.45
CLAM	**0.66** ± **0.10**	0.44	0.43

## Data Availability

The data presented in this study are available in the GDC Portal at https://portal.gdc.cancer.gov/.

## Abbreviations

The following abbreviations are used in this manuscript:

ABMIL	Attention-Based Multiple Instance Learning
AUC	Area Under the Curve
CLAM	Clustering-Constrained Attention Multiple Instance Learning
CV	Cross Validation
DFTD	Dual-Feature Tokenization Multiple Instance Learning
DEG	Differentially Expressed Gene
FDR	False Discovery Rate
GDC	Genomic Data Commons
GO	Gene Ontology
H&E	Hematoxylin and Eosin
HCC	Hepatocellular Carcinoma
MIL	Multiple Instance Learning
MLP	Multi-Layer Perceptron
mRMR	Minimum Redundancy Maximum Relevance
MVI	Microvascular Invasion
ROC	Receiver Operating Characteristic
ROI	Region of Interest
ROS	Reactive Oxygen Species
SSM	Single Somatic Mutation
SVM	Support Vector Machine
TCGA	The Cancer Genome Atlas
TransMIL	Transformer-based Multiple Instance Learning
WSI	Whole-Slide Image
WSL	Weakly Supervised Learning
